# Supplementary material for: Actin-microtubule cytoskeletal interplay mediated by MRTF-A/SRF signaling promotes dilated cardiomyopathy caused by LMNA mutations
Source: Nat Commun. 2022 Dec 22;13:7886. doi: 10.1038/s41467-022-35639-x (PMC9780334; doi:10.1038/s41467-022-35639-x)
Supplement: Supplementary file 1 — Supplementary Information [file 41467_2022_35639_MOESM1_ESM.pdf]

Actin-microtubule cytoskeletal interplay mediated by MRTF-A/SRF signaling promotes dilated cardiomyopathy caused by *LMNA* mutations

Supplementary information

C. Le Dour[#], M. Chatzifrangkeskou[#], C. Macquart[#], M.M. Magiera, C. Peccate, C. Jouve, L. Virtanen, T. Heliö, K. Aalto-Setälä, S. Crasto, B. Cadot, D. Cardoso, N. Mougenot, D. Adesse, E. Di Pasquale, J.S. Hulot, P. Taimen, C. Janke, A. Muchir[*]

[#] These authors contributed equally to this work.

[*] Correspondence to
Antoine Muchir, Centre de Recherche en Myologie, Sorbonne Université, INSERM UMRS974, Faculté de Médecine La Pitié-Salpêtrière, 105 boulevard de l'Hôpital, 75013 Paris – France; email: a.muchir@institut-myologie.org.

SUPPLEMENTARY FIGURES

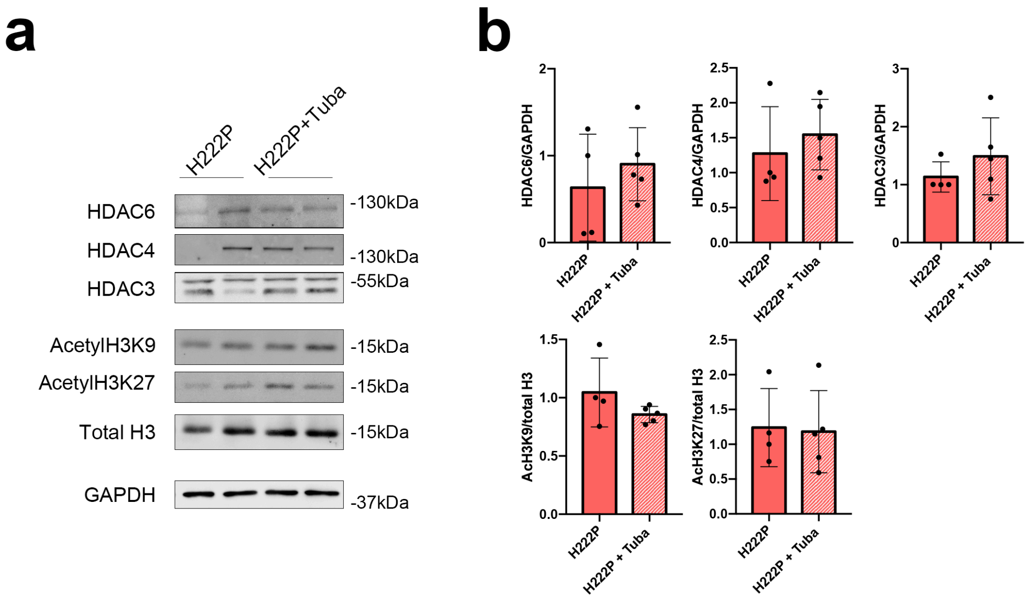

Figure S1| Tubastatin A treatment has no effect on HDAC expression and acetylation of histone H3. (a) Representative immunoblots showing expression of histone deacetylases: HDAC6, HDAC4, HDAC3, and acetylation levels of histone H3 (AcH3K9 and AcH3K27) in hearts from *Lmna*$^{p.H222P/H222P}$ mice (H222P) treated with tubastatin A (+Tuba) as compared with treated with DMSO. **(b)** Bar graph showing quantification of expression of HDAC6, HDAC4 and HDAC3 normalized to GAPDH and expression of acetylated H3K9 and H3K27 normalized to total H3 in hearts from *Lmna*$^{p.H222P/H222P}$ (H222P) mice treated with tubastatin A (+Tuba) (n=5) compared to mice treated with DMSO (n=4) (mean ± SD). Source data are provided as a Source Data file.

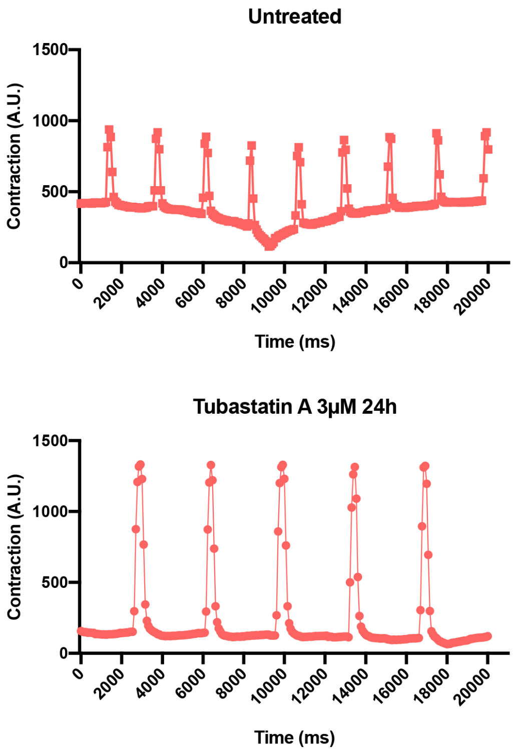

Figure S2| Tubastatin A treatment ameliorates contraction profile of iPS-CM carrying *LMNA* p.H222P mutation. Representative contraction profile obtained after analysis with MuscleMotion ImageJ macro of iPS-CM carrying *LMNA* p.H222P mutation, cultured in monolayer, matured for 40 days post differentiation and spontaneously beating, treated (lower panel) or not (upper panel) with tubastatin A 3μM for 24h.

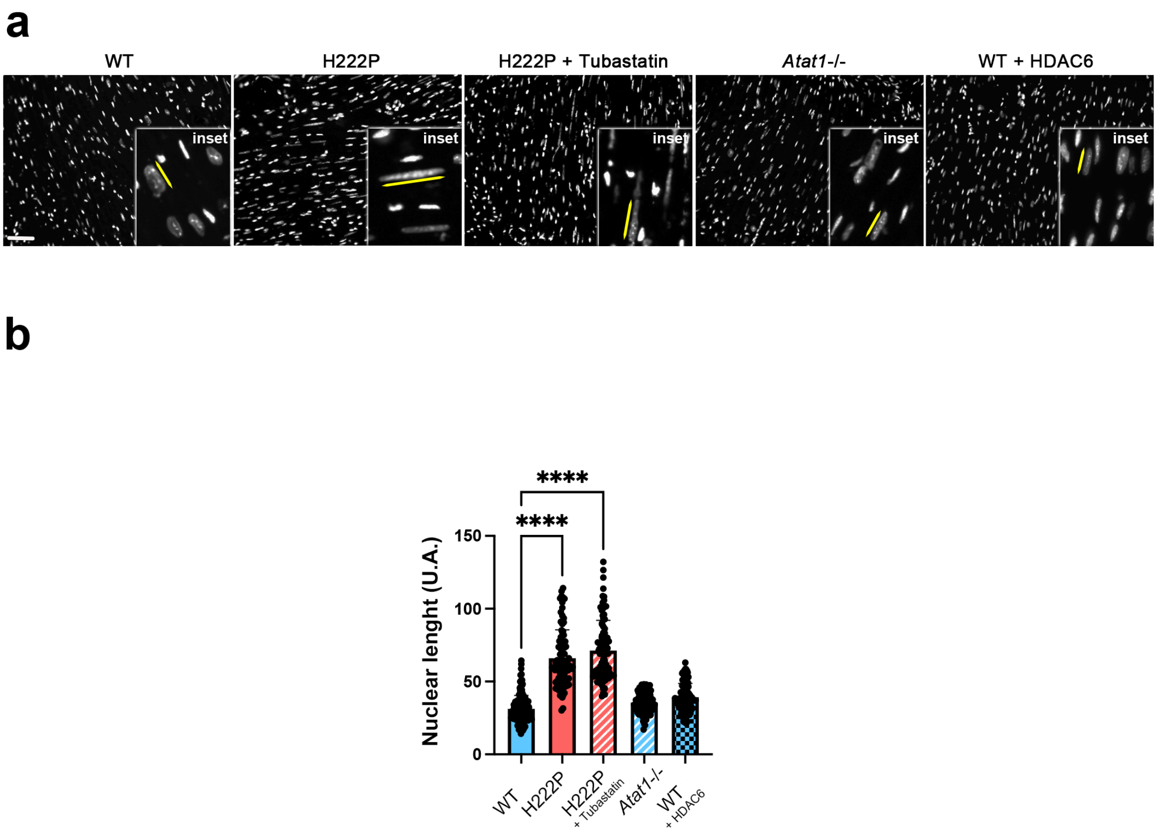

Figure S3| α-Tubulin acetylation is not affecting nuclear shape. (a) Representative micrographs showing the nucleus labeling (dapi staining) of heart sections from WT mice and *Lmna*$^{p.H222P/H222P}$ mice, *Lmna*$^{p.H222P/H222P}$ mice treated with tubastatin A, *Atat1*$^{-/-}$ mice and WT mice transduced with AAV-HDAC6. Scale bar: 50 µm. **(b)** Graph showing the quantification of cardiac nuclear length from cardiomyocytes from the different mouse models (n=170 WT, n=90 H222P, n=92 H222P + Tubastatin A, n=117 *Atat1*$^{-/-}$, n=131 WT+HDAC6 nuclei, mean ± SD), One-Way ANOVA followed by Tukey's multiple comparison test, ****p≤0.0001. Source data are provided as a Source Data file.

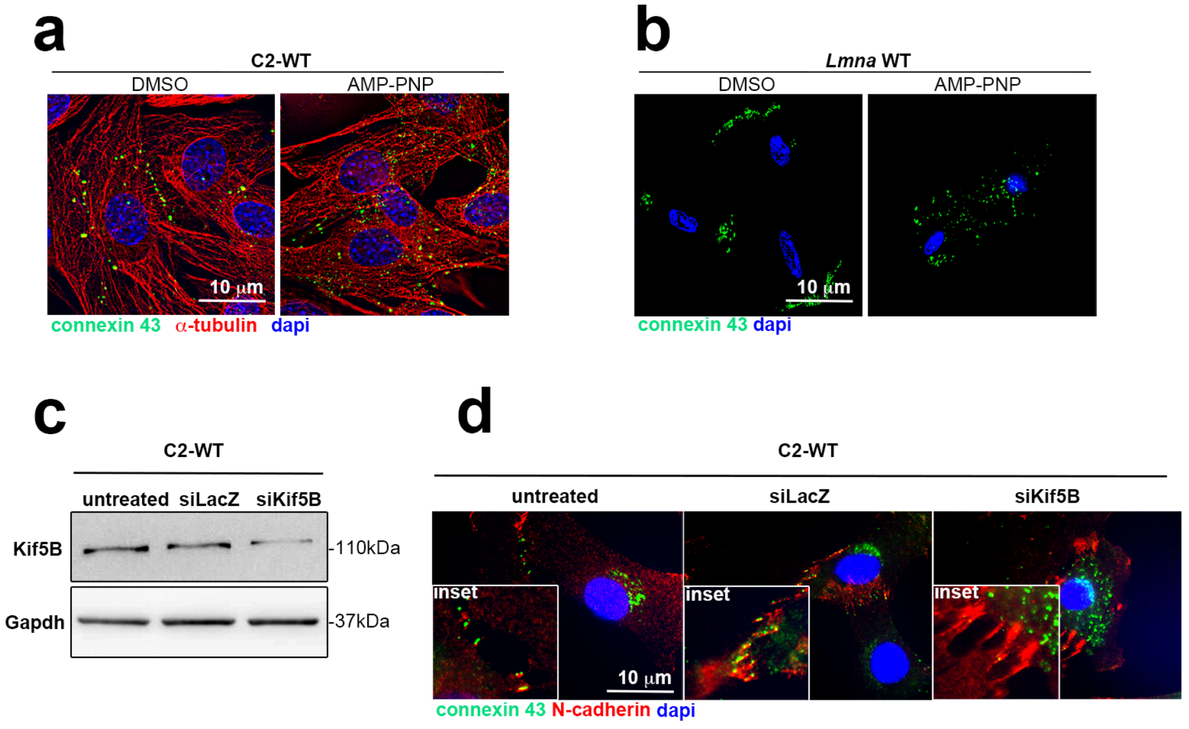

Figure S4| Cx43 localization depends on kinesin motor proteins. (a) Fluorescence micrographs showing Cx43 and α-actinin labeling of C2-WT cells. Nuclei counter-stained with dapi are also shown. **(b)** Fluorescence micrographs showing Cx43 labeling of isolated adult cardiomyocytes from WT mice. Nuclei counter-stained with dapi are also shown. **(c)** Immunoblot showing Kif5B expression from C2-WT cells treated or not with siRNA against Kif5B. **(d)** Fluorescence micrographs showing Cx43 and N-cadherin labeling of C2-WT cells treated or not with siRNA against Kif5B. Nuclei counter-stained with dapi are also shown. **(a,b,c,d)** A representative of three independent repeats is shown. Source data are provided as a Source Data file.

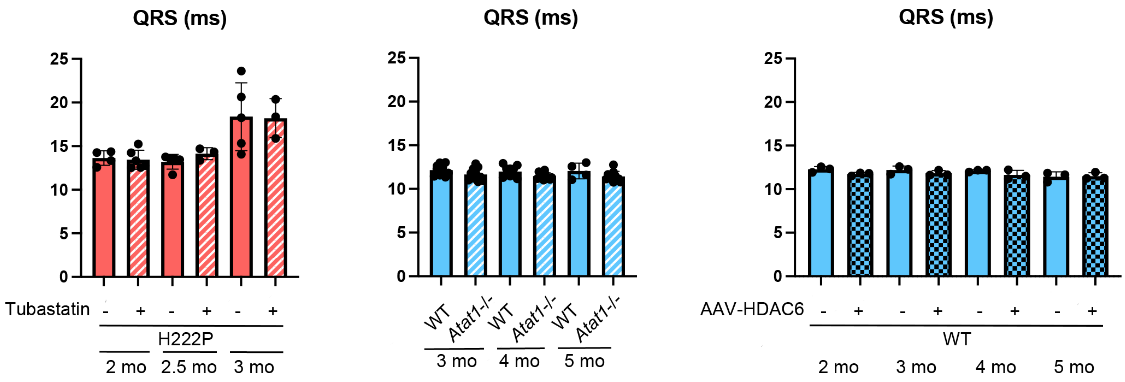

Figure S5| α-Tubulin acetylation is not affecting intraventricular conduction. Graph showing QRS interval from (left panel) *Lmna*$^{p.H222P/H222P}$ mice treated with tubastatin A from 2 (n=6) to 3 (n=3) month of age as compared with DMSO-treated (n=5), (middle panel) *Atat1*$^{-/-}$ mice at 3, 4 and 5 month of age (n=11) as compared with WT mice (n=10) and (right panel) WT mice transduced with AAV-HDAC6 (n=3) as compared with PBS-injected mice (n=3). Results are means ± SEM.

SUPPLEMENTARY TABLES

Table S1| Echocardiographic data for *Lmna*$^{+/+}$ (WT) mice transduced or not transduced with AAV-cofilin-1 or AAV-cofilin-1-T25A at 6 months of age. IVS, inter ventricular septum; LVD, left ventricular diameter; LVPW, left ventricular posterior wall; LVED, left ventricular end diastolic; LVES, left ventricular end systolic; EF, ejection fraction; FS, fractional shortening; s, systole; d, diastole. Values are means ± SEM. *p≤0.05, **p≤0.01 and ***p≤0.001 between *Lmna*$^{+/+}$ mice transduced and *Lmna*$^{+/+}$ mice not transduced. Values for *Lmna*$^{p.H222P/H222P}$ mice are shown as comparison.

	***Lmna* WT**<br>**NT**	***Lmna* WT**<br>**AAV-cofilin WT**	***Lmna* WT**<br>**AAV-cofilin T25A**	***Lmna* H222P**<br>**NT**
**age**	6 months	6 months	6 months	6 months
**n**	3	3	3	3
**time (ms)**	93,033 ± 1,86	101,82 ± 2,37 **	108,13 ± 1,66 ***	96,11 ± 2,88
**heart rate (bpm)**	645,07 ± 12,80	589,53 ± 13,61 **	554,9 ± 8,6 ***	624,6 ± 18,51
**cardiac output (l/min)**	0,06 ± 0	0,05 ± 0,01	0,06 ± 0,02	0,07 ± 0,01
**IVSd (cm)**	0,07 ± 0	0,06 ± 0,005	0,07 ± 0,005	0,06 ± 0
**LVDd (cm)**	0,35 ± 0,01	0,36 ± 0,03	0,37 ± 0,04	0,47 ± 0,07 *
**LVPWd (cm)**	0,07 ± 0	0,06 ± 0	0,07 ± 0,011	0,06 ± 0,005
**IVSs (cm)**	0,12 ± 0,01	0,09 ± 0,01 **	0,13 ± 0,005	0,09 ± 0,01 **
**LVDs (cm)**	0,18 ± 0,005	0,23 ± 0,025	0,19 ± 0,025	0,37 ± 0,09 **
**LVPWs (cm)**	0,12 ± 0,01	0,09 ± 0,005	0,11 ± 0,015	0,08 ± 0 **
**LVED vol (ml)**	0,113 ± 0,005	0,12 ± 0,026	0,13 ± 0,04	0,26 ± 0,11
**LVES vol (ml)**	0,02 ± 0	0,036 ± 0,01	0,02 ± 0,01	0,14 ± 0,10
**EF (%)**	84,4 ± 0,58	71,12 ± 3,67 *	84,28 ± 1,58	48,760 ± 16,50 **
**FS (%)**	47,32 ± 0,67	35,01 ± 2,89 *	47,22 ± 1,68	21,54 ± 9,27 ***
**LV eject vol (ml)**	0,09 ± 0,005	0,08 ± 0,015	0,11 ± 0,03	0,11 ± 0,02
**h/r**	0,39 ± 0,007	0,34 ± 0,032	0,40 ± 0,03	0,26 ± 0,03 **

Table S2| Top scoring gene ontology (Biological Functions) in hearts from *Lmna*$^{+/+}$ (WT) mice transduced or not transduced with AAV-cofilin-1 or AAV-cofilin-1-T25A at 6 months of age. Values for *Lmna*$^{p.H222P/H222P}$ mice are shown as comparison.

The most statistically significant functions identified are listed according to their p value. In red are biological functions related to cytoskeleton.

Diseases and Biological Functions	*p*-value		
	Lmna H222P NT	*Lmna* WT AAV-cofilin WT	*Lmna* WT AAV-cofilin T25A
Cell movement of muscle cells	2,47E-13	3,71E-09	N.S.
Endothelial cell development	1,44E-07	9,86E-09	N.S.
Migration of muscle cells	4,28E-13	1,39E-08	N.S.
Organization of fibrils	2,05E-10	1,73E-06	N.S.
Organization of filaments	3,44E-09	1,76E-06	N.S.
Morphology of filaments	5,64E-08	4,94E-06	N.S.
Morphology of skeleton	1,86E-06	1,38E-05	N.S.
Cell spreading	1,11E-10	2,52E-05	N.S.
Fibrosis	1,20E-14	3,68E-05	N.S.
Accumulation of macrophages	5,14E-07	6,52E-05	N.S.
Organization of actin cytoskeleton	3,73E-10	1,17E-04	N.S.
Cell movement of fibroblast cell lines	1,06E-09	1,44E-04	N.S.
Migration of endothelial cells	2,51E-07	1,54E-04	N.S.
Formation of filopodia	1,05E-07	1,55E-04	N.S.
Fibrogenesis	1,35E-12	1,58E-04	N.S.
Activation of antigen presenting cells	7,97E-09	1,66E-04	N.S.
Differentiation of connective tissue cells	1,58E-11	1,76E-04	N.S.
Cell movement of connective tissue cells	2,80E-08	2,85E-04	N.S.
Quantity of connective tissue	5,27E-13	6,04E-04	N.S.
Cell-cell contact	1,80E-08	7,45E-04	N.S.
Organization of cytoskeleton	7,40E-17	7,66E-04	N.S.
Morphology of connective tissue cells	3,24E-08	8,62E-04	N.S.
Cell viability	2,52E-08	1,27E-03	N.S.
Quantity of connective tissue cells	3,59E-10	1,37E-03	N.S.
Cell movement of fibroblasts	4,46E-09	1,74E-03	N.S.
Morphology of heart ventricle	2,69E-07	1,85E-03	N.S.
Formation of filaments	1,63E-10	2,22E-03	N.S.
Migration of antigen presenting cells	4,23E-11	4,88E-03	N.S.
Binding of fibroblasts	3,25E-08	6,85E-03	N.S.
Cellular infiltration	8,81E-19	7,27E-03	N.S.
Attachment of cells	1,74E-12	7,97E-03	N.S.
Quantity of adipose tissue	3,57E-07	8,31E-03	N.S.

Table S3| Top scoring gene ontology (Canonical Pathways) in hearts from *Lmna*$^{+/+}$ (WT) mice transduced or not transduced with AAV-cofilin-1 or AAV-cofilin-1-T25A at 6 months of age. Values for *Lmna*$^{p.H222P/H222P}$ mice are shown as comparison.

The most statistically significant canonical pathways identified are listed according to their p value. In red are the canonical pathways of interest in this study.

Canonical Pathways	*p*-value		
	Lmna H222P NT	*Lmna* WT AAV-cofilin WT	*Lmna* WT AAV-cofilin T25A
**ERK/MAPK Signaling**	**1,53E-02**	**3,20E-04**	**N.S.**
Leukocyte Extravasation Signaling	4,34E-05	4,78E-04	N.S.
Oxidative Ethanol Degradation III	6,61E-03	5,88E-04	N.S.
Dendritic Cell Maturation	2,94E-03	6,14E-04	N.S.
Fatty Acid α-oxidation	3,53E-02	6,88E-04	N.S.
Putrescine Degradation III	1,12E-02	7,97E-04	N.S.
Tryptophan Degradation X (Mammalian, via Tryptamine)	3,23E-03	9,17E-04	N.S.
Ethanol Degradation IV	4,27E-03	1,05E-03	N.S.
Phospholipases	1,68E-02	1,60E-03	N.S.
Ethanol Degradation II	1,52E-04	1,88E-03	N.S.
Noradrenaline and Adrenaline Degradation	3,93E-04	2,53E-03	N.S.
VEGF Signaling	1,16E-02	2,59E-03	N.S.
Integrin Signaling	1,81E-05	2,79E-03	N.S.
Gαq Signaling	7,45E-03	2,80E-03	N.S.
Phospholipase C Signaling	2,30E-02	3,17E-03	N.S.
Axonal Guidance Signaling	2,53E-04	3,43E-03	N.S.
Phagosome Formation	1,94E-08	3,67E-03	N.S.
Fc Epsilon RI Signaling	2,10E-02	4,54E-03	N.S.
Role of NFAT in Regulation of the Immune Response	1,74E-02	5,02E-03	N.S.
IL-17 Signaling	1,62E-02	7,36E-03	N.S.
Fcγ Receptor-mediated Phagocytosis in Macrophages and Monocytes	2,39E-04	8,28E-03	N.S.
VEGF Family Ligand-Receptor Interactions	2,83E-02	9,27E-03	N.S.
Serotonin Degradation	1,99E-02	1,03E-02	N.S.
**Actin Cytoskeleton Signaling**	**1,94E-05**	**1,35E-02**	**N.S.**
FAK Signaling	4,85E-03	1,45E-02	N.S.
Superpathway of Inositol Phosphate Compounds	8,79E-03	1,55E-02	N.S.
p38 MAPK Signaling	2,76E-02	1,59E-02	N.S.
Apelin Cardiac Fibroblast Signaling Pathway	1,27E-05	1,59E-02	N.S.
Alanine Biosynthesis II	8,99E-03	1,76E-02	N.S.
Alanine Degradation III	8,99E-03	1,76E-02	N.S.
Signaling by Rho Family GTPases	2,50E-02	2,17E-02	N.S.
Sphingosine-1-phosphate Signaling	8,95E-03	2,18E-02	N.S.
ILK Signaling	8,77E-09	2,47E-02	N.S.
Role of Pattern Recognition Receptors in Recognition of Bacteria and Viruses	4,79E-03	2,75E-02	N.S.
Glioma Invasiveness Signaling	1,63E-06	3,07E-02	N.S.
Regulation of Actin-based Motility by Rho	5,62E-04	3,60E-02	N.S.
Inhibition of Matrix Metalloproteases	3,37E-06	4,21E-02	N.S.
Macropinocytosis Signaling	1,41E-06	4,29E-02	N.S.
Granulocyte Adhesion and Diapedesis	6,41E-04	4,86E-02	N.S.
PKCθ Signaling in T Lymphocytes	3,60E-02	4,86E-02	N.S.

Table S4| Top scoring Upstream Regulators in hearts from *Lmna*$^{+/+}$ (WT) mice transduced or not transduced with AAV-cofilin-1 or AAV-cofilin-1-T25A at 6 months of age. Values for *Lmna*$^{p.H222P/H222P}$ mice are shown as comparison.

The most statistically significant upstream regulators identified are listed according to their p value. In red is the upstream regulator of interest in this study.

Upstream Regulators	*p*-value		
	Lmna H222P NT	*Lmna* WT AAV-cofilin WT	*Lmna* WT AAV-cofilin T25A
INHBB	2,96E-06	4,45E-08	N.S.
ITGB6	4,44E-05	3,11E-06	N.S.
EGR1	2,55E-09	3,39E-06	N.S.
RETNLB	1,59E-10	5,42E-06	N.S.
C3AR1	2,85E-04	5,89E-06	N.S.
SPTLC2	1,06E-06	6,20E-06	N.S.
MAP3K1	1,86E-05	8,30E-06	N.S.
TBX5	2,80E-02	8,30E-06	N.S.
GLIS2	2,20E-08	1,11E-05	N.S.
C5AR1	2,04E-05	3,40E-05	N.S.
CTGF	8,27E-06	4,24E-05	N.S.
SEMA7A	1,98E-07	4,28E-05	N.S.
ADA	5,22E-03	5,15E-05	N.S.
ERBB4	8,22E-08	2,92E-04	N.S.
CYR61	6,13E-04	3,48E-04	N.S.
ERBB3	9,30E-13	3,62E-04	N.S.
DCN	1,86E-06	4,27E-04	N.S.
KLF4	1,79E-07	6,89E-04	N.S.
BMP2	5,69E-03	1,44E-03	N.S.
TGFB2	1,94E-04	1,58E-03	N.S.
SPARC	2,29E-03	1,86E-03	N.S.
SRC	2,42E-04	2,46E-03	N.S.
TNFRSF1A	2,78E-03	3,63E-03	N.S.
TLR3	2,66E-03	3,95E-03	N.S.
SOX4	1,72E-04	4,32E-03	N.S.
HMGA1	1,05E-03	4,53E-03	N.S.
RELA	3,96E-05	4,57E-03	N.S.
TNFRSF1B	1,70E-02	5,38E-03	N.S.
JUN	5,08E-06	6,35E-03	N.S.
**SRF**	**1,04E-02**	**8,97E-03**	**N.S.**
STAT3	1,75E-12	1,04E-02	N.S.
EGF	2,15E-06	1,06E-02	N.S.
CDKN2A	2,72E-02	1,65E-02	N.S.
WNT3A	3,50E-05	2,22E-02	N.S.
OGA	5,05E-05	2,37E-02	N.S.
STAT6	1,12E-06	3,92E-02	N.S.
SHH	1,05E-02	4,02E-02	N.S.
ACOX1	8,43E-07	4,20E-02	N.S.

Table S5| SRF target genes regulation in hearts from *Lmna*$^{+/+}$ (WT) mice transduced or not transduced with AAV-cofilin-1 or AAV-cofilin-1-T25A at 6 months of age. Values for *Lmna*$^{p.H222P/H222P}$ mice are shown as comparison. (green: down-regulation, red: up-regulation).

EntrezGeneID	Gene	Description	Fold		
			***Lmna* H222P NT**	***Lmna* WT AAV-cofilin WT**	***Lmna* WT AAV-cofilin T25A**
12721	*CORO1A*	coronin 1A	-1.07837	-1.08182	1.00244
56431	*DSTN*	destrin, actin depolymerizing factor	-1.46583	-1.11139	1.02303
11475	*ACTA2*	actin, alpha 2, smooth muscle, aorta	-1.14666	-1.3521	1.02515
22003	*Tpm1*	tropomyosin 1, alpha	-1.06179	-1.01997	1.01253
59091	*JPH2*	junctophilin 2	-1.1997	-1.03441	1.1358
14200	*FHL2*	four and a half LIM domains 2	-2.51028	-1.64494	1.0339
17929	*MYOM1*	myomesin 1	-1.22636	-1.07403	1.02004
225115	*SVIL*	supervillin	-1.06002	-1.1449	1.02517
17698	*MSN*	moesin	-2.23618	-1.25362	1.17429

Table S6| ECG parameters. Baseline values of ECG parameters measured in *Lmna*$^{p.\mathrm{H222P/H222P}}$ mice treated with Tubastatin A as compared with DMSO-treated (top table); in *Atat1*$^{-/-}$ (KO) mice as compared with wild-type mice (WT) (middle table) and in mice transduced with AAV overexpressing HDAC6 as compared with WT mice (bottom table).

	**H222P**	**H222P**	**H222P**	**H222P**	**H222P**	**H222P**
	**DMSO**	**Tubastatin**	**DMSO**	**Tubastatin**	**DMSO**	**Tubastatin**
**age**	2 months	2 months	2,5 months	2,5 months	3 months	3 months
**n**	4	5	4	3	4	3
RR (ms)	80.55 ± 1.9	80.84 ± 0.9	80.3 ± 1.2	78.9 ± 0.6	78.61 ± 1.1	79.3 ± 0.8
PR (ms)	34.23 ± 0.7	35.1 ± 0.6	33.7 ± 0.6	32.82 ± 0.77	34.08 ± 0.7	32.55 ± 0.38
QRS (ms)	13.64 ± 0.42	13.44 ± 0.44	13.20 ± 0.37	14.136 ± 0.39	18.38 ± 1.7	18.20 ± 1.29

	**WT**	**Atat1 KO**	**WT**	**Atat1 KO**	**WT**	**Atat1 KO**
**age**	3 months	3 months	4 months	4 months	5 months	5 months
**n**	10	11	10	11	4	11
RR (ms)	80.55 ± 1.9	80.84 ± 0.9	80.3 ± 1.2	78.9 ± 0.6	78.61 ± 1.1	79.3 ± 0.8
PR (ms)	34.23 ± 0.7	35.1 ± 0.6	33.7 ± 0.6	32.82 ± 0.77	34.08 ± 0.7	32.55 ± 0.38
QRS (ms)	12.16 ± 0.19	11.66 ± 0.2	11.99 ± 0.19	11.46 ± 0.11	12.05 ± 0.44	11.43 ± 0.17

	**WT**	**AAV-HDAC6**	**WT**	**AAV-HDAC6**	**WT**	**AAV-HDAC6**	**WT**	**AAV-HDAC6**
**age**	2 months	2 months	3 months	3 months	4 months	4 months	5 months	5 months
**n**	3	3	3	3	3	3	3	3
RR (ms)	78.8 ± 0.32	79.72 ± 0.46	78.77 ± 0.8	81.58 ± 1.3	79.20 ± 1.6	83.11 ± 3.4	83.05 ± 1.6	86.14 ± 4.7
PR (ms)	33.2 ± 0.7	33.24 ± 0.7	32.67 ± 0.37	34.16 ± 0.9	33.15 ± 1.58	34.56 ± 1.2	36.168 ± 1.07	34.41 ± 1.14
QRS (ms)	12.27 ± 0.18	11.74 ± 0.11	12.20 ± 0.26	11.81 ± 0.18	12.11 ± 0.07	11.63 ± 0.31	11.44 ± 0.32	11.49 ± 0.23

Table S7| Clinical parameters of patients whose iPS-derived cardiomyocytes were used in this study.

LMNA mutation	Age	Sex	Diagnosis	Echocardiography	Electrocardiography	Reference
p.S143P	30-35	F	DCM	DCM	1st degree AV block and paroxysmal flutter	Kärkkäinen et al., 2004; West et al., 2016; Shah et al., 2019
p.R190W	35-40	M	DCM	DCM	ventricular ectopic beats	Salvarani et al. 2009
p.H222P	20-25	M	EDMD	normal	arrhythmias	Bonne et al. 2000

Table S8| Listing of primers used in this study

Gene	Forward	Reverse
Srf	5' cccaccacagaccagagaat 3'	5' agttggtgatggggaaggag
Acta2	5' aggaaccctgagacgctgct 3'	5' ccattccaaccattactccc 3'
Fhl2	5' acgagacctgcttcacctgt 3'	5' tgctcccggtaagtaacacc 3'
Myom1	5' gccctcagaaacgactgaag 3'	5' gagcagtcgtcagtccatga 3'
Svil	5' gccctacaagaagctcatgc 3'	5' ccaagctccctctttgtctg 3'
Tnnc1	5' gacggtgacaagaacaacga 3'	5' ggaatggggagagaaagtcg 3'
Msn	5' tggagcagcacaaactcaac 3'	5' atgttgagacccaaggcatc 3'
Rplp0	5' ctccaagcagatgcagcaga 3'	5' atagccttgcgcatcatggt 3'
Atat1	5' ggaacaggaagcggaggata 3'	5' ctggcgttcattatgtcccc 3'
