# Supplementary material for: Actin-microtubule cytoskeletal interplay mediated by MRTF-A/SRF signaling promotes dilated cardiomyopathy caused by LMNA mutations
Source: Nat Commun. 2022 Dec 22;13:7886. doi: 10.1038/s41467-022-35639-x (PMC9780334; doi:10.1038/s41467-022-35639-x)
Supplement: Supplementary file 3 — Reporting Summary [file 41467_2022_35639_MOESM3_ESM.pdf]

nature portfolio

Corresponding author(s): Antoine Muchir

Last updated by author(s): 2022/11/23

Reporting Summary

Nature Portfolio wishes to improve the reproducibility of the work that we publish. This form provides structure for consistency and transparency in reporting. For further information on Nature Portfolio policies, see our Editorial Policies and the Editorial Policy Checklist.

Statistics

For all statistical analyses, confirm that the following items are present in the figure legend, table legend, main text, or Methods section.

n/a	Confirmed	
[ ]	[x]	The exact sample size (*n*) for each experimental group/condition, given as a discrete number and unit of measurement
[ ]	[x]	A statement on whether measurements were taken from distinct samples or whether the same sample was measured repeatedly
[ ]	[x]	The statistical test(s) used AND whether they are one- or two-sided<br>*Only common tests should be described solely by name; describe more complex techniques in the Methods section.*
[x]	[ ]	A description of all covariates tested
[ ]	[x]	A description of any assumptions or corrections, such as tests of normality and adjustment for multiple comparisons
[ ]	[x]	A full description of the statistical parameters including central tendency (e.g. means) or other basic estimates (e.g. regression coefficient) AND variation (e.g. standard deviation) or associated estimates of uncertainty (e.g. confidence intervals)
[ ]	[x]	For null hypothesis testing, the test statistic (e.g. *F*, *t*, *r*) with confidence intervals, effect sizes, degrees of freedom and *P* value noted<br>*Give P values as exact values whenever suitable.*
[x]	[ ]	For Bayesian analysis, information on the choice of priors and Markov chain Monte Carlo settings
[x]	[ ]	For hierarchical and complex designs, identification of the appropriate level for tests and full reporting of outcomes
[x]	[ ]	Estimates of effect sizes (e.g. Cohen's *d*, Pearson's *r*), indicating how they were calculated

Our web collection on statistics for biologists contains articles on many of the points above.

Software and code

Policy information about availability of computer code

Data collection	ImageLab version 2.4 software was used for data collection from western blots membranes.<br>Metamorph version 7.10 software was used to collect microscopy raw images.<br>Affymetrix GeneChip and GeneTraffic 3.0 (Stratagene) software was used to generate image files from Affymetrix microarray.<br>SparkControl software was used to quantify luminescence upon luciferase assay.<br>Electrocardiograms waveforms were recorded using Iox Software.<br>Transthoracic echocardiography data were collected with Vivid 7 System software.<br>Autodeblur v9.1 was used for immunofluorescence images deconvolution.
Data analysis	FIJI/ImageJ2 software was used for quantification of western blots and signal analysis in microscopy.<br>MucleMotion open macro on FIJI/ImageJ2 was used to determine contraction profiles of cardiomyocytes.<br>GraphPad Prism 8 software was used for statistical analysis.<br>ErmineJ (version 2.1.12; http://www.bioinformatics.ubc.ca/ermineJ/) was used for functionnal analysis of gene expression data.<br>LightCycler 480 software version 1.5.0 was used for RT-qPCR quantifications.<br>Electrocardiograms waveforms were analyzed with ECG Auto software

For manuscripts utilizing custom algorithms or software that are central to the research but not yet described in published literature, software must be made available to editors and reviewers. We strongly encourage code deposition in a community repository (e.g. GitHub). See the Nature Portfolio guidelines for submitting code & software for further information.

Data

Policy information about availability of data

All manuscripts must include a data availability statement. This statement should provide the following information, where applicable:

- Accession codes, unique identifiers, or web links for publicly available datasets
- A description of any restrictions on data availability
- For clinical datasets or third party data, please ensure that the statement adheres to our policy

All data generated or analysed during this study are included in this published article (and its supplementary information files). The Affymetrix microarray data generated in this study have been deposited in Gene Expression Omnibus database under accession code GSE218891.

Human research participants

Policy information about studies involving human research participants and Sex and Gender in Research.

Reporting on sex and gender	No sex and gender analysis were performed
Population characteristics	The subjects were a 23-year-old man with cardiomyopathy associated with muscular dystrophy carrying LMNA p.delK261 mutation, a 53-year-old man with cardiomyopathy carrying LMNA p.E33D mutation and a 47-year-old woman with cardiomyopathy carrying LMNA p.R60G mutation. Control human heart samples were obtained from a 57-year-old man with an intracranial bleed, a 15-year-old woman who died of a drug overdose and a 46-year-old man who died from end-stage liver disease
Recruitment	Sections of explanted hearts from human subjects with LMNA mutations were obtained without identifiers from Myobank-AFM de l'Institut de Myologie.
Ethics oversight	Myobank-AFM received approval from the French Ministry of Health and from the Committee for Protection of Patients to share tissues and cells of human origin for scientific purposes, ensuring the donors' anonymity, respect of their volition and consent according to the legislation.

Note that full information on the approval of the study protocol must also be provided in the manuscript.

Field-specific reporting

Please select the one below that is the best fit for your research. If you are not sure, read the appropriate sections before making your selection.

☒ Life sciences ☐ Behavioural & social sciences ☐ Ecological, evolutionary & environmental sciences

For a reference copy of the document with all sections, see nature.com/documents/nr-reporting-summary-flat.pdf

Life sciences study design

All studies must disclose on these points even when the disclosure is negative.

Sample size	Sample sizes were determined based on established practice and according to accepted standards in the field (at least 3 independent experiments in vitro and at least 3 mice for each group). These sample sizes have been shown to be sufficient to evaluate effects of Tubastatin A (Osseni, A et al., 2022; d'Ydewalle, C et al, 2011). Individual data points are shown in each figure or described in the figure legend. Microarray analysis was performed with 3 individuals as it is typical for transcriptomic analysis.All experiments were repeated at least three times independently to ensure reproducibility.
Data exclusions	No data were excluded from analysis
Replication	All experiments were repeated three times, unless indicated in the figure legend or methods. All attempts at replication were successful and actual n values are indicated in the text for all experiments.
Randomization	All mice were randomly assigned to groups. For cell culture experiments, cells were split, plated, and then treated with DMSO or molecules in a randomized manner.
Blinding	Many of the experiments were independently conducted by multiple authors. Echocardiographer and electrocardiographer were blinded regarding the genotype and the treament of mice. All image processing studies were analyzed in a blinded-manner by Zoheir Guesmia.

Reporting for specific materials, systems and methods

We require information from authors about some types of materials, experimental systems and methods used in many studies. Here, indicate whether each material, system or method listed is relevant to your study. If you are not sure if a list item applies to your research, read the appropriate section before selecting a response.

Materials & experimental systems

n/a	Involved in the study
	☒ Antibodies
	☒ Eukaryotic cell lines
☒	☐ Palaeontology and archaeology
	☒ Animals and other organisms
☒	☐ Clinical data
☒	☐ Dual use research of concern

Methods

n/a	Involved in the study
☒	☐ ChIP-seq
☒	☐ Flow cytometry
☒	☐ MRI-based neuroimaging

Antibodies

Antibodies used

The following antibodies were used:
pERK1/2 Cell Signaling #9101 (dilution 1/500); ERK1/2 Cell Signaling #4696 (dilution 1/500); Phospho-Cofilin-1 (Ser3) Cell Signaling #3311 (dilution 1/500); Cofilin-1 Cell Signaling 5175(dilution 1/500); Sarcomeric Alpha Actinin Sigma A7811 (dilution 1/500); Acetyl-tubulin alpha1a Sigma T7451 (dilution 1/500); tubulin alpha abcam ab18251 (dilution 1/500); GAPDH abcam ab8245 (dilution 1/500); Connexin 43 abcam ab11370 (dilution 1/500); FLAG M2 sigma F3165 (dilution 1/500); Actin Cytoskeleton AAN01 (dilution 1/500); MRTF-A Santa Cruz sc-398675 (dilution 1/200); Emerin Novocastra #NCL (dilution 1/500); N-Cadherin (D4R1H) Cell Signaling #13116 (dilution 1/500); Troponin T (Cardiac) Cell Signaling #5593 (dilution 1/500); HDAC6 (D21B10) Cell Signaling #7612 (dilution 1/500); Phospho-Cofilin-1 (Thr25) GenScript Custom (dilution 1/50); Alexa fluor 568 goat anti-mouse IgG, Invitrogen # A-11004 1/1000, Alexa fluor 488 goat anti-rabbit IgG, Invitrogen # A-11008 1/1000, Alexa fluor 488 donkey anti-goat IgG Invitrogen # A-11055 1/1000, StarBright Blue 520 Goat Anti-Mouse IgG, Biorad #12005866 1/10,000, StarBright Blue 700 Goat Anti-Rabbit IgG, Biorad #12004162 1/10,000

Validation

Validation statements for all antibodies used were supplied by the manufacturer for the species and specification. Information on this validation can be found on the manufacturer's website for each antibody listed. In detail:

pERK1/2 Cell Signaling #9101/ reactivity: H,M,R / apllications: WB, IF, IP / https://www.cellsignal.com/products/primary-antibodies/phospho-p44-42-mapk-erk1-2-thr202-tyr204-antibody/9101
ERK1/2 Cell Signaling #4696 / reactivity: H,M,R / apllications: WB, IF / https://www.cellsignal.com/products/primary-antibodies/p44-42-mapk-erk1-2-l34f12-mouse-mab/4696
Phospho-Cofilin-1 (Ser3) Cell Signaling #3311 / reactivity: H,M,R / apllications: WB / https://www.cellsignal.com/products/primary-antibodies/phospho-cofilin-ser3-antibody/3311
Cofilin-1 Cell Signaling 5175 / reactivity: H,M,R apllications: WB,IF / https://www.cellsignal.com/products/primary-antibodies/cofilin-d3f9-xp-rabbit-mab/5175?_=1558518620661&Ntt=5175&tahead=true
Sarcomeric Alpha Actinin Sigma A7811 / reactivity: H,M,R apllications: WB,IF / https://www.abcam.com/sarcomeric-alpha-actinin-antibody-ea-53-ab9465.html
Acetyl-tubulin alpha1a Sigma T7451 / reactivity: H,M,R apllications: WB,IF / https://www.sigmaaldrich.com/FR/fr/product/sigma/t7451
tubulin alpha abcam ab18251 / reactivity: H,M,R apllications: WB,IF / https://www.abcam.com/alpha-tubulin-antibody-microtubule-marker-ab18251.html
GAPDH abcam ab8245 / reactivity: H,M,R apllications: WB,IF / https://www.abcam.com/gapdh-antibody-6c5-loading-control-ab8245.html
Connexin 43 abcam ab11370 / reactivity: H,M,R apllications: WB,IF / https://www.abcam.com/connexin-43-gja1-antibody-ab11370.html
FLAG M2 sigma F3165 / reactivity: H,M,R apllications: WB,IF / https://www.sigmaaldrich.com/catalog/product/sigma/f3165?lang=fr&region=FR
Actin Cytoskeleton AAN01 / reactivity: H,M,R apllications: WB,IF / https://www.cytoskeleton.com/aan01/
MRTF-A Santa Cruz sc-398675 / reactivity: H,M,R apllications: WB,IF,IP / https://www.scbt.com/p/mrtf-a-antibody-g-7?requestFrom=search
Emerin Novocastra NCL/ reactivity: H,M,R apllications: WB,IF / https://www.labome.com/product/Leica-Biosystems/NCL-EMERIN.html
N-Cadherin (D4R1H) Cell Signaling #13116 / reactivity: H,M,R apllications: WB,IF / https://www.cellsignal.com/products/primary-antibodies/n-cadherin-d4r1h-xp-rabbit-mab/13116
Troponin T (Cardiac) Cell Signaling #5593 / reactivity: H,M,R apllications: WB,IF,IP,ChIP / https://www.cellsignal.com/products/primary-antibodies/troponin-t-cardiac-antibody/5593?site-search-type=Products&N=4294956287&Ntt=ctnt&fromPage=plp&_requestid=171794
HDAC6 (D21B10) Cell Signaling #7612 / reactivity: H,M apllications: WB,IP / https://www.cellsignal.com/products/primary-antibodies/hdac6-d21b10-rabbit-mab/7612

Anti-phospho(T25)-cofilin-1 (Genscript) was custom made. Upon overexpression and knock-down of phospho(T25)-cofilin-1, we validated the antibody in the lab by band specificity and product size in western blot experiments.

Eukaryotic cell lines

Policy information about cell lines and Sex and Gender in Research

Cell line source(s)	C2C12# CRL-1772; HEK293 #CRL-1573 cell line were obtained from the American Type Culture Collection (ATCC).
Authentication	None of the cell lines used were authenticated
Mycoplasma contamination	All cell lines were tested negative for mycoplasma.
Commonly misidentified lines (See ICLAC register)	No cell line used are commonly misidentified

Animals and other research organisms

Policy information about studies involving animals; ARRIVE guidelines recommended for reporting animal research, and Sex and Gender in Research

Laboratory animals	Transgenic LmnaH222P/H222P and Atat1 knock-out male mice from 2 to 6 month were bred on a C57BL/6J background. Control littermates were used. Mice were housed in pathogen-free facility, 12h:12h light/dark cycles, controled temperature of 22°C, and 55% humidity, with normal activity, standard chow diet (7% simple sugars, 3% fat, 50% polysaccharide, 15% protein (w/w)) and water ad libitum.
Wild animals	The study did not involve wild animals.
Reporting on sex	All experiments were carried out using male mice as cardiac phenotype occurs earlier and is more severe than in female mice (Arimura et al., Hum Mol Genet, 2005).
Field-collected samples	None used
Ethics oversight	All animal experiments were approved by the French Ministry of Health at the Center for Research in Myology for the Care and Use of Experimental Animals. The animal experiments were performed according to the guidelines from Directive 2010/63/EU of the European Parliament regarding the protection of animals used for scientific purposes.

Note that full information on the approval of the study protocol must also be provided in the manuscript.
